# Antagonism of NF-κB-up-regulated micro RNAs (miRNAs) in sporadic Alzheimer's disease (AD)—anti-NF-κB vs. anti-miRNA strategies

**DOI:** 10.3389/fgene.2013.00077

**Published:** 2013-05-01

**Authors:** Walter J. Lukiw

**Affiliations:** Department of Neuroscience and Ophthalmology, LSU Neuroscience Center, Louisiana State University Health Sciences CenterNew Orleans, LA, USA

**Figure 1:**
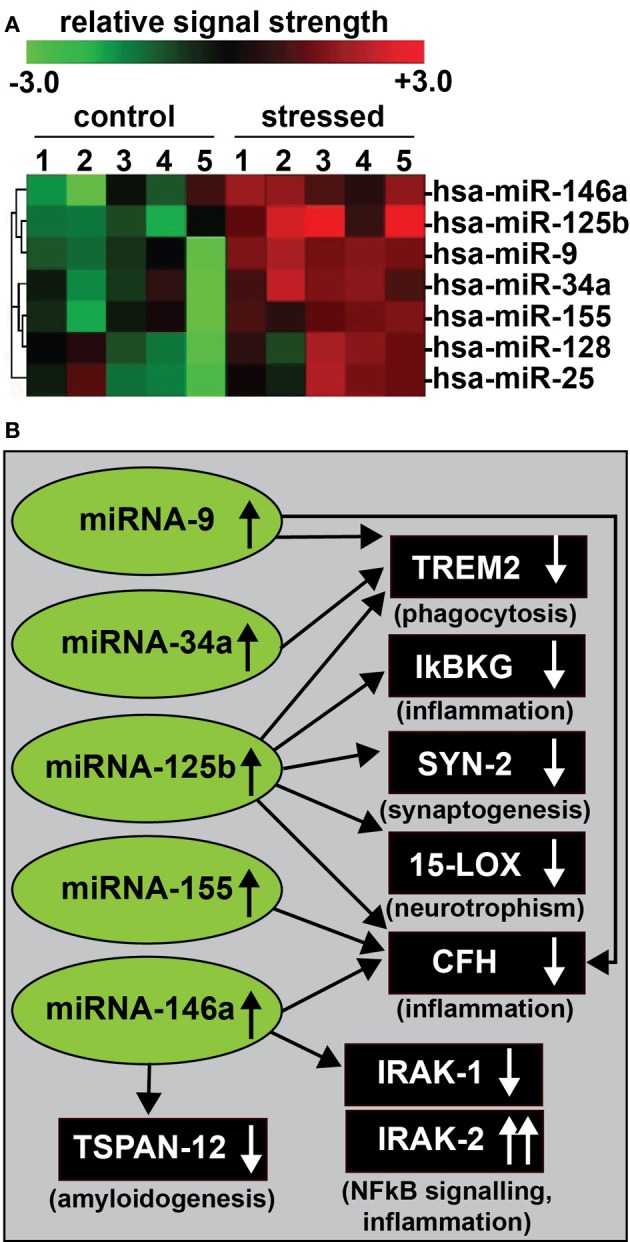
**(A)** In recent experiments human primary neuronal-glial (HNG) cells were treated with an AD-relevant, NF-κ B-inducing cocktail of amyloid beta 42 (Aβ 42) and interleukin-1beta (IL-1β), and inducible miRNAs were analyzed using miRNA arrays (Lukiw, 2012a). Confirmation of miRNA induction and NF-κ B sensitivity was obtained (1) using LED-Northern dot blot and/or RT-PCR analysis; (2) by inhibition of this induction using specific NF-κ B inhibitors CAPE, CAY10512, and PDTC and (3) by bioinformatics analysis of functional NF-κ B binding sites in the promoters of the genes that encode these inducible miRNAs (Lukiw et al., 2008; Cui et al., 2010; Lukiw, 2012a). A small family of 5 miRNAs—*miRNA-9, miRNA-34a, miRNA-125b, miRNA-146a, and miRNA-155*—appear to be up-regulated in high quality total RNA isolated from short post-mortem AD brains; note that *hsa-miR-128 and miR-25* are variably up-regulated; *N* = 6; **(B)** These findings in part define a highly interactive network of NF-κ B-sensitive, up-regulated miRNAs in stressed human brain cells and AD hippocampus that can explain much of the observed neuropathology in AD including deficits in phagocytosis (TREM2), innate-immune signaling and chronic inflammation (IkBKG, CFH, IRAK-1, and IRAK-2), impairments in neurotransmitter packaging and release (SYN-2), neurotrophism (15-LOX), and amyloidogenesis (TSPAN12) (see references in text); these up-regulated miRNAs and down-regulated mRNAs form a highly integrated, pathogenic miRNA-mRNA signaling network resulting in gene expression deficits in sporadic AD that may be self-perpetuating due to chronic re-activation of NF-κB stimulation via IRAK-2 pathways (Cui et al., 2010; Lukiw, 2012a,b,c). Inhibition of the NF*-κB initiator or* individual blocking of the pathogenic induction of these five miRNAs may provide novel therapeutic approaches for the clinical management of AD, however, what NF-κ B or miRNA inhibition strategies, or whether they can be utilized either alone or in combination, remain open to question. Preliminary data has indicated that these approaches may neutralize this chronic, inducible, progressive pathogenic gene expression program to re-establish brain cell homeostasis, and ultimately be of novel pharmacological use in the clinical management of AD.

As research into micro RNA (miRNA) speciation, complexity, and biological activity progresses, it is becoming clear that a selective subset of all so far characterized miRNAs utilized by human cells and tissues is under the transcriptional control of the pro-inflammatory and immune system-linked transcription factor NF-κB (Sen and Baltimore, 1986; Lukiw and Bazan, 1998; Taganov et al., 2006; Lukiw, 2007, 2012a,b,c; Bazzoni et al., 2009; Ma et al., 2011; Boldin and Baltimore, 2012; Cremer et al., 2012; Li et al., 2012; Lukiw and Alexandrov, 2012; Zhao et al., 2013). The inducible up-regulation of NF-κ B-sensitive miRNAs is by virtue of single, and often multiple, NF-κB-DNA binding recognition sites in the regulatory regions of miRNA-containing genes that drive RNA polymerase II-mediated transcription of pre-miRNA species (Ambros, 2004; Taganov et al., 2006; Baltimore et al., 2008; Cui et al., 2010; Guo et al., 2010; Bredy et al., 2011; Lukiw, 2012a). In neurodegenerative disease research, stress-triggered up-regulation of these NF-κ B-induced miRNAs appear to be playing pathogenic roles in the down-regulation of brain-essential messenger RNAs (mRNA), and the initiation and propagation of pathological gene expression programs that are, for example, characteristic of the Alzheimer's disease (AD) process (Figure 1). NF-κB-mediated up-regulated miRNAs and down-regulated mRNA targets thereby form a highly integrated, pathogenic NF-κ B-miRNA-mRNA signaling network that can explain much of the observed neuropathology in AD, including deficits in phagocytosis (Niemitz, 2012; Zhao et al., 2013), NF-κ B-mediated innate-immune signaling and chronic inflammation (Cui et al., 2010; Heneka et al., 2010; Lukiw and Bazan, 2010; Lukiw et al., 2012), impairments in neurotransmitter packaging and release, neurotrophism and amyloidogenesis (Xu et al., 2009; Lukiw, 2012a,b,c). Under homeostatic conditions, NF-κ B activation involves a coordinated, sequential, and self-limiting sequence of events controlled by positive and negative regulatory mechanisms, however, this does not appear to be the case in early, moderate or especially advanced stages of sporadic AD. In sporadic AD, once initiated, NF-κ B-mediated disruption of homeostatic gene expression can be self-perpetuating due, in part, to the chronic re-activation of NF-κ B activities via up-regulation of interleukin-1β receptor associated kinase-2 (IRAK-2) signaling pathways (Cui et al., 2010). Selective inhibition of the actions of NF-κ B and specific NF-κ B-sensitive miRNAs therefore seems a plausible therapeutic strategy towards neutralizing their combined effects in sporadic AD, and related progressive, age-related neurological diseases with an innate-immune and inflammatory component.

The brain-ubiquitous transcription factor NF-κ B comprises a family of heterodimeric DNA-binding proteins, for example the relatively common p50–p65 complex, that normally reside in a “resting state” in the cytoplasm (Sen and Baltimore, 1986; Lukiw and Bazan, 1998; Mattson and Camandola, 2001; Yamamoto and Gaynor, 2001; Lukiw, 2012a,b). In general, cytoplasmic NF-κ B activation is stimulated via a wide array of physiological stressors including ionizing radiation, viral infection, neurotoxic metals, elevations in reactive oxygen species (ROS), inflammatory cytokines and chemokines, Aβ 42 peptides, hypoxia, and other forms of physiological stress (Baltimore et al., 2008; Pogue et al., 2009, 2011; Boldin and Baltimore, 2012). This activation is largely mediated by the serine- phosphorylation of a family of NF-κB inhibitory subunits, non-covalently bound to the NF-κ B heterodimer, and collectively known as the inhibitors of kappaB (Iκ B). Iκ B phosphorylation is followed, in turn, by Iκ B degeneration, in part through cooperation with NF-κ B essential modulator (NEMO) proteins (Baltimore et al., 2008; Shifera, 2010). NF-κ B heterodimers next translocate to the nucleus to target binding sites homologous to the canonical DNA sequence 5′-GGGACTTTCC-3′ in the regulatory regions of NF-κ B-sensitive genes (Meffert and Baltimore, 2005; Wong et al., 2011). Ever since NF-κ B's original description in 1986 several hundred NF-κ B inhibitors, both natural and synthetic, have been developed to inhibit NF-κ B-activation in this complex signaling network (Sen and Baltimore, 1986; Mattson and Camandola, 2001; Aggarwal and Shishodia, 2004; Meffert and Baltimore, 2005; Gilmore and Herscovitch, 2006; Nam, 2006; Gilmore and Garbati, 2011). Indeed, inhibition of NF-κ B can involve multifaceted aspects of NF-κ B activation via blocking of NF-κB signaling at various control points, from inhibition of the kinases that phosphorylate Iκ B (thus preventing activation of NF-κ B), to the deacetylation of the NF-κ B p65 subunit, to the proteasome-mediated degradation of the Iκ B, to the translocation of NF-κ B to the nucleus, to the prevention of NF-κ B binding to DNA recognition sites using decoy and/or antisense strategies (Meffert and Baltimore, 2005; Gilmore and Herscovitch, 2006; Nam, 2006; Yang et al., 2007; Gilmore and Garbati, 2011; Lukiw et al., 2012). Importantly, constitutive NF-κ B signaling is critically homeostatic to many aspects of normal brain function, and essential to the control of cell proliferation, apoptosis, innate, and adaptive immunity, the inflammatory response and related stress responses. NF-κ B activation is also an important part of a cellular recovery process that may protect brain cells against oxidative-stress or brain trauma-induced apoptosis and induced neurodegeneration, hence antagonism of NF-κ B may reduce its intrinsic potential for neuroprotective activity (Lukiw and Bazan, 1998; Yang et al., 2007; Lukiw, 2012a,b,c). Since NF-κ B may have conflicting effects in different brain cell types it is important to assess anti-NF-κ B (or anti-miRNA) inhibition strategies in individual cell types that constitute susceptible tissues. For example, pro-inflammatory NF-κ B-sensitive miRNAs such as miRNA-146a have been shown to be of varying basal abundance, and be variably induced, in different human primary brain and retinal cell types (Alexandrov et al., 2011; Li et al., 2011). Another interesting example of interactive NF-κ B and miRNA effects are the differential regulation of the interleukin-1 receptor associated kinases IRAK-1 and IRAK-2 through NF-κ B actions (Cui et al., 2010; Flannery and Bowie, 2010). In the regulation of IRAK-1 expression, NF-κ B has been shown to induce miRNA-146a which, by virtue of a miRNA-146a recognition feature in the IRAK-1 3′-UTR, down-regulates IRAK-1 expression while at the same time activating transcription of IRAK-2, by virtue of NF-κ B binding in the IRAK-2 upstream 5′ regulatory region (Cui et al., 2010). Importantly the IRAK-1 gene is devoid of NF-κ B recognition features in its 5′ regulatory region, while the IRAK-2 3′-UTR has no recognition feature for miRNA-146a. Hence anti-NF-κ B strategies may affect the pleiotrophic regulation of expression of even highly homologous genes, such as IRAK-1 and IRAK-2 that function in the innate immune response, the sensing of pathogens and the initiation of immunity.

An analogous example of the perceived perils of wide-spectrum NF-κ B inhibition can be taken from neurooncology, and the broad therapeutic strategies of using the common alkylating anti-neoplastic drug temozolamide (TMZ). TMZ attaches an alkyl group to the guanine base of DNA, resulting in base pair stabilization or cross-linking, the inhibition of cell growth and stimulation of apoptosis, and has been widely used to treat glioblastoma and other cancers (Thomas et al., 2013). As the guanines of all genomic DNA are susceptible to alkylation, the use of TMZ is tolerated because high rates of cell proliferation renders cancer cells more sensitive to alkyl modification. However, the guanines of other cell DNAs that are naturally rapidly dividing may also be alkylated leading to off-target neurotoxic and neurological effects (Lai et al., 2008; Nagasawa et al., 2012; Thomas et al., 2013). Similarly, global anti-NF-κ B therapeutic strategies may be more effective when NF-κ B is widely over stimulated in highly progressive neuropathological situations such as AD (Lukiw and Bazan, 1998; Yang et al., 2007; Lukiw, 2013). Put another way, NF-κB-inhibition strategies may be maximized only when NF-κ B stimulated effects have surpassed a critical threshold that are en masse seriously detrimental to the normal homeostatic function of brain cells and tissues. However, it is still problematic and pharmacologically difficult to minimize the complicating effects of NF-κ B inhibition on essential, low-level homeostatic NF-κ B function (Nam, 2006; Lukiw, 2012b).

On the other hand, anti-miRNA (antagomir, AM) approaches are much more target-efficient and a stabilized anti-ribonucleotide, anti-miRNA sequence 100% homologous, and complementary to the target miRNA will inhibit that target miRNA 95% or more with extremely high efficacy (Lukiw et al., 2008; Cui et al., 2010). Interestingly, tailoring imperfections into base-pair complementarity between the anti-miRNA ribonucleotides and the target miRNA could result in partial inhibition of the miRNA with a tailored “throttle-down” of miRNA-mediated effects, and could be used as a therapeutic strategy of “variable efficacy.” It is clear that the appropriate clinical application of these anti-NF-κ B or anti-miRNA strategies requires a more intimate understanding of the specific NF-κ B and miRNA mechanisms responsible for their activation pathway in different brain cell types, definition of the optimal point of intervention in the NF-κ B or miRNA activation pathway, and the characterization of potent and specific inhibitors of the chosen NF-κ B or miRNA targets. Another formidable challenge lies in the implementation of innovative anti-NF-κ B and anti-miRNA strategies without incurring prohibitive off-target toxicity from combinatorial approaches. Indeed, as has been found from cancer research, rigorous anti-NF-κ B strategies may have long term health consequences and appropriate cautions should be exercised (Gilmore and Herscovitch, 2006; Gilmore and Garbati, 2011; Nagasawa et al., 2012; Thomas et al., 2013).

Lastly, virtually each day is now providing increased insight into the induction of NF-κ B and miRNA in response to physiological stressors, including the mechanisms that regulate the bioavailability of pro-inflammatory transcription factors and miRNAs in immune health and disease. The inducers of NF-κ B also appear to be the inducers of a highly selective sub-family of NF-κ B-sensitive miRNAs (Lukiw, 2012a,b,c). These in turn drive the expression of a characteristic AD phenotype that includes an up-regulation in inflammatory signaling, amyloidogenesis, impairment in microglial cell-mediated phagocytosis, and deficits in synaptic and neurotrophic signaling (Figure 1). Because NF-κ B can variably regulate gene expression directly by transcriptional activation and indirectly by miRNA-mediated post-transcriptional down-regulation it will be important to further map out specific NF-κ B-miRNA-mRNA signaling pathways to ascertain at what point anti-NF-κ B or anti-miRNA-based strategies may be best prescribed to fit the individual pathological situation. While NF-κ B up-regulation helps define a subset of inducible NF-κ B-sensitive pre-miRNA genes, there may be other transcription factors, or combinations of transcription factors that define the induction of other small families of pre-miRNAs. Hence a general rule to follow may be that a highly interactive network of NF-κ B or other transcription factor-sensitive up-regulated miRNAs in stressed human brain cells define an epigenetic expression program that is amenable to strategic manipulation at some point, to eventually normalize the dynamic balance between inducible neurodegeneration or cell survival signals.
